# Negative symptoms in alcohol use disorder: A pilot study applying the two-factor model of negative symptoms to patients with alcohol use disorder

**DOI:** 10.3389/fpsyt.2022.957924

**Published:** 2022-11-18

**Authors:** Maximilian Buschner, Kenneth M. Dürsteler, Gina Fischli, Jelena Hess, Matthias Kirschner, Stefan Kaiser, Marcus Herdener

**Affiliations:** ^1^Center for Addictive Disorders, Department of Psychiatry, Psychotherapy, and Psychosomatics, Psychiatric Hospital, University of Zurich, Zurich, Switzerland; ^2^Clinic for Adult Psychiatry, University Psychiatric Clinics, University of Basel, Basel, Switzerland; ^3^Division of Adult Psychiatry, Department of Psychiatry, Geneva University Hospitals, Geneva, Switzerland

**Keywords:** alcohol use disorder, negative symptoms, anhedonia, craving, substance use disorder, addiction, motivation and pleasure

## Abstract

**Background and aims:**

Alcohol Use Disorder (AUD) is characterized by a reduction in goal-directed behavior, with alcohol use taking precedence over other areas of life. These features in AUD resemble negative symptoms in schizophrenia, especially the reduction in motivation and pleasure (MAP). Given the clinical similarities of negative symptoms across diagnostic categories, it comes as a surprise that there are few investigations on negative symptoms in alcohol and other substance use disorders. To our knowledge, our study is the first to assess negative symptoms in AUD based on a two-factorial approach, and to investigate the interrelation of these dimensions with the severity of AUD, and alcohol craving.

**Materials and methods:**

We examined a sample of 42 patients with AUD at the Psychiatric University Hospital in Zurich. Participants provided self-report and interview-based measures of the severity of AUD, negative symptoms, and alcohol craving. Finally, we used data from the electronic health records of the patients.

**Results:**

Patients with AUD show negative symptoms to a similar extent as patients with schizophrenia or bipolar disorder. We found a positive correlation between the extent of impairment within the MAP factor and overall severity of AUD. Furthermore, MAP negative symptoms were correlated with alcohol craving. In a linear regression, negative symptoms predicted alcohol craving whereas depression did not.

**Summary:**

Negative symptoms as conceptualized for schizophrenia are prevalent in patients with AUD and associated with the severity of AUD. More specifically, severity of AUD correlates with diminished motivation and pleasure, highlighting the importance of disturbances in motivational functions in AUD. This is further supported by the correlation between negative symptoms and craving, a hallmark of AUD. Taken together, our findings suggest that negative symptoms might be a highly relevant but hitherto often neglected therapeutic target in AUD.

## Introduction

Alcohol is extensively used worldwide (1). Besides its desired acute effects, like euphoria and anxiolysis, excessive alcohol use has negative health consequences. Chronic alcohol use is among the leading causes for premature death and contributes to the global burden of neuropsychiatric and somatic diseases with enormous direct and indirect economic costs (2, 3). An estimated 4.3% of the Swiss population aged over 15 years show a chronic pattern of risky alcohol consumption (4). Lifetime prevalence of alcohol use disorder (AUD) is estimated to be 8.6% (1). Of those suffering from AUD, over 80% do not receive adequate treatment (5). After treatment, relapse is common (6). Monahan and Finney found abstinence rates of only 43% after treatment (7). Even after achieving long-term abstinence, there seems to be an annual relapse rate of 3% (8).

Among the features of AUD are substance craving, and a shift in goal-directed behavior toward the obtainment and use of alcohol (9). The upcoming ICD-11 considers this shift in behavior as one of the three main features that characterize alcohol dependence: “*Substance use becomes an increasing priority in life such that its use takes precedence over other interests or enjoyments, daily activities, responsibilities, or health or personal care. Substance use takes an increasingly central role in the person’s life and relegates other areas of life to the periphery*…” (10).

During the course of AUD and other substance use disorders (SUDs), substance use progresses from an initially voluntary to a more habitual and finally obsessive-compulsive stage (11). The brain’s reward system is profoundly dysregulated in addictive disorders and plays a key role in the development and maintenance of addiction (12–14). The adaptations affect different neurotransmitter systems including dopamine (15–17), glutamate (18–20), and GABA (21). In animal addiction models, different motivational states within the cycle of substance-seeking are paralleled by distinct oscillations in synaptic strength within the pathway between the prefrontal cortex and the nucleus accumbens (22), two important hubs for reward processing (23). Also in human imaging studies, functioning of those regions have been significantly altered in individuals with SUDs, indicated by increased activity in response to substance-related cues (24–26), which is linked to increased substance craving (27, 28). In contrast, the prefrontal cortex, and the nucleus accumbens show reduced responsiveness toward naturally rewarding cues such as social stimuli and monetary reinforcers (29–31).

In schizophrenia, the symptoms nowadays termed negative symptoms (32) have been considered a hallmark of the disease since Kraepelin and Bleuler (33, 34). As defined by the National Institute of Mental Health Measurement and Treatment Research to Improve Cognition in Schizophrenia (NIMH-MATRICS) Consensus Statement, negative symptoms include the following domains: blunted affect, alogia, asociality, anhedonia, and avolition (35). These domains can be summarized in two factors; the first, “motivation and pleasure” (MAP, sometimes referred to as “apathy”), consists of the domains asociality, anhedonia, and avolition. (36–38). The second factor, “diminished expression” (DIM), includes the domains blunted affect and alogia. The neurobiological basis for deficits in the motivation and pleasure domain is still debated; however, areas involved in reward prediction, like the ventral striatum, may be central (39). Negative symptoms are also present in patients with schizophrenia and comorbid SUD (40–42).

Anhedonia, which is defined as a reduced experience of pleasure is regarded as a core feature of schizophrenia and is also a key symptom of depression and common in various other psychiatric conditions (43). Recent research has shown that patients with schizophrenia, however, often report a normal or even elevated hedonic response to reward (44, 45). Their ability to *anticipate* pleasure in future reward, on the other hand, is diminished (46, 47). These patients show a social performance rather than a hedonic deficit (37). This has led to a distinction between anticipatory (“wanting”) and consummatory (“liking”) anhedonia (48). Interestingly, this distinction was first conceptualized in SUDs (17).

In SUDs, anhedonia has been regarded as part of the (prolonged) withdrawal symptomatology (49–54), a possible risk factor for relapse (55, 56) and as crucial for treatment outcome (57–59). Nguyen et al. found that anhedonia correlated with relapse rates in AUD (60). In studies in patients with cocaine use disorder, anhedonia had a negative impact on the effectiveness of contingency management treatment (57, 61, 62). A study by Huhn et al. in patients recovering from opioid use disorder showed a reduced activation of the prefrontal cortex for natural reward that in association with the extent of anhedonia (58). Janiri and colleagues found a significant correlation between anhedonia and substance craving (63). Furthermore, an anhedonic trait has been discussed as a risk factor in the development of addiction (64–66). For a systematic review of the literature on anhedonia in substance use disorders, see Garfield et al. (67).

The other two domains that comprise the motivation and pleasure factor of negative symptoms are asociality and avolition (35). Asociality can be defined as a lack of motivation to engage in social interaction. Avolition is a general reduction in the ability to initiate goal-directed behavior. In summary, the factor “motivation and pleasure” describes different aspects of an inability to anticipate and engage in behaviors usually regarded as pleasurable or otherwise rewarding. This factor shows a great degree of similarity with two of the diagnostic criteria of AUD in Diagnostic and Statistical Manual of Mental Disorders, Fifth Edition (DSM-5) (9):

– A great deal of time is spent in activities necessary to obtain alcohol, use alcohol, or recover from its effects.
– Important social, occupational, or recreational activities are given up or reduced because of alcohol use.

The second factor, “diminished expression,” signifies the reduced capability to experience and/or express emotions. The subdimension “blunted affect” refers to the subjective experience and non-verbal expression of emotions, whereas “alogia” means poverty of verbal expression (35).

Whereas anhedonia, in particular trait and consummatory anhedonia, has been studied in populations with SUDs, to our knowledge no study has yet applied a more extended model of negative symptoms to AUD. Considering the similarities between negative symptoms in schizophrenia and some of the clinical features in AUD, it seems plausible to examine whether the full spectrum of negative symptoms—not just consummatory anhedonia—can be found in patients with AUD (68).

In this pilot study, we examined the two-factorial model of negative symptoms in a sample of patients with AUD. We further investigated whether MAP or DIM are specifically related to overall severity of AUD and craving.

## Materials and methods

### Study setting

This cross-sectional study was conducted at the Psychiatric University Hospital Zurich, Switzerland. Clinical interviews and assessments took place from July 2020 until January 2021.

The studies involving human participants were reviewed and approved by Cantonal Ethics Committee, Zurich, Switzerland. The participants provided their written informed consent to participant in this study.

### Sample

Prior to the start of the study, all therapists at the Center for Addictive Disorders and the Center for Integrative Psychiatry at the Psychiatric University Hospital Zurich (Psychiatrische Universitätsklinik Zürich, PUK) were asked to check for eligible patients.

Inclusion criteria for study participation were as follows: diagnosis of AUD regardless of the stage of the disorder (e.g., abstinent, currently addicted, relapsed), age between 18 and 65 years, ability to provide written informed consent and to communicate in German. Exclusion criteria were a current or former diagnosis of schizophrenia or bipolar disorder, a diagnosis of severe neurological disorders or other somatic disorders which would impact the ability to participate. All other comorbidities were allowed.

Sixty-three eligible patients were reported to us by their respective therapist, 14 of whom did not respond to our contact *via* telephone, six refused to participate, and one patient did not meet the inclusion criteria. Therefore, the final sample consisted of 42 patients in total, 33 of whom were outpatients and nine were inpatients. Ten participants have been abstinent from alcohol for a miminum of 30 days prior to their inclusion in the study. For details, see Table 1.

**TABLE 1:** Demographic and sample characteristics.

Characteristic	M	*N*	%	SD	Range
Female		18	42.9		
Age (years)	43.74			10.61	22–65
Inpatients		9	21.4		
Number of inpatients stays	5.19			7.14	0–34
No inpatient stays		7	16.7		
1–3 inpatient stays		20	47.6		
4–10 inpatient stays		6	14.3		
More than 10		9	21.4		
Suicidality (light or severe)		15	35.7		
Diagnoses					
Major depression		12	28.6		
No comorbidities		8	19.0		
One comorbid disorder		13	31.0		
More than one comorbidity besides AUD		20	47.6		
PSP score (psychosocial functioning)	2.64			0.70	1.3–4.5
Cognitive Variables					
Digit Symbol Substituion Test (*n* in 120 s)	57.72			14.66	14–90
Letter-Number Sequencing (longest letter-number sequence)	8.98			2.93	3–14

Inpatients stays refer to the stays at PUK. Diagnoses were collected with the MINI and psychosocial functioning with the Personal and Social Performance Scale (PSP). Cognitive functioning was measured with the Digit Symbol Substitution Test (DSST) and the Letter-Number Sequencing. The MINI data is missing for one participant.

### Measures

#### Clinical interviews and questionnaires

The Mini International Neuropsychiatric Interview (MINI) is a structured diagnostic interview consisting of up to 120 questions that allows for diagnosing axis-I disorders of the DSM-IV as well as suicidality (69). It is a structured, easy to conduct interview requiring only minimal training.

The Center for Epidemiological Studies Depression Scale (CES-D) in its German Version (Allgemeine Depressionsskala, ADS-L) was used to assess depressive symptoms (70, 71). The ADS-L is a 20-item self-report questionnaire, items are rated on a four-point Likert scale from 0 (rarely/not at all) to 4 (most of the time). A score of 23 or higher indicates clinically relevant depressive symptoms.

The Calgary Depression Scale for Schizophrenic Patients (CDSS) was developed to assess depressive symptoms in patients with schizophrenia in distinction from negative and extrapyramidal symptoms (72, 73). It has been validated in patients with major depressive disorder (74) as well as healthy subjects (75). It is a semi-structured interview consisting of nine items. The first eight items are open-ended questions; the interviewer rates participants’ answers on a four-point Likert scale ranging from severe to absent. For the last item, the interviewer rates the extent of depressive symptoms observed during the interview. A cut-off score of six allows for identification of depression in patients with schizophrenia.

Substance use was recorded using a Timeline Followback (TLFB) form. Any alcohol use was defined as drinking alcohol on a minimum of 3 days per week. For the last 7 days, the number of drinking days and the number of alcoholic beverages per drinking day were recorded. Currently abstinent participants were asked to name the number of alcoholic beverages usually consumed in 1 week. Harmful use of alcohol was defined as drinking more than one standard drink per day for women and two standard drinks for men, respectively.

The German Version of the Obsessive Compulsive Drinking Scale (OCDS-G) (76) is a self-assessment scale consisting of 18 questions. It captures cognitive aspects such as preoccupation with alcohol consumption, the amount of alcohol consumed typically, the subjective extent of substance craving, psychosocial impairments following alcohol consumption and the feeling of control over alcohol consumption. It also includes three visual analog scales on which participants rate the extent of craving.

The Brief Negative Symptoms Scale (BNSS) is a semi-structured 13-item interview on six domains, namely the five domains of negative symptoms defined by the NIMH MATRICS Consensus Definition Conference plus lack of normal distress as a sixth domain (77). These domains are assigned to two dimensions, diminished expression (DIM) and motivation and pleasure (MAP). The MAP dimension consists of the three domains, anhedonia, avolition and asociality, whereas the two domains, affective flattening and alogia (poverty of speech), form the DIM dimension. The interviewer asks open-ended questions regarding social and other activities as well as stressful events and rates the extent of impairment on a seven-point Likert scale.

The Self Evaluation of Negative Symptoms (SNS) is a 20-item questionnaire for the self-assessment of the five domains of negative symptoms blunted affect, alogia, social withdrawal, anhedonia, and avolition (78). Each item is rated on a 3-point Likert scale. The sum of all 20 items forms a total score, ranging from 0 to 40 corresponding to the severity of negative symptoms. A score below seven is considered non-pathological.

The Temporal Experience of Pleasure (TEPS) (79) measures anticipatory and consummatory hedonic capacity and consists of 18 items on a 6-point Likert scale. The average of the score of each item forms the total score with higher scores indicating higher hedonic capacity and lower scores indicating higher anhedonia, respectively. It has been tested in a sample with opioid-dependent participants (80).

To assess cognitive functioning, we used the Digit Symbol Substitution Test (DSST) and Letter-Number Sequencing, two subtests of the Wechsler Adult Intelligence Scale(81).

#### Therapist-rated questionnaires

The Rapid Addiction Profile (RAP) is rated by the therapist on a 4-point scale (82). It covers five dimensions: somatic level, psychiatric level, motivation level, crisis level and resource level. The total score ranges from 0 to 20 points, with higher scores indicating greater severity of AUD.

The Personal and Social Performance Scale (PSP) (83) is rated by the participants’ therapists to assess the level of impairment of social dysfunction during the last 30 days. The scale covers four areas of social functioning, namely socially useful activities such as occupation and study, personal and other social relationships, self-sufficiency, and aggressive or otherwise disturbing behavior. The level of dysfunction in each area is rated on a 6-point Likert scale.

#### Statistical analysis

According to the MINI interview, three participants did not fully meet diagnostic criteria for AUD. For each case, we contacted their therapist and re-evaluated the results of the MINI interview together with them. For all three patients we could ensure that diagnostic criteria for AUD were, in fact, fulfilled. One participant had been abstinent for approximately 13 months prior to the interview, formally being considered as remitted. Since the patient was still in outpatient treatment for AUD and, at the time of the first contact with the study personnel, still fulfilled the criteria for AUD, they were included in the study. One patient could only partially conduct the interview; his missing data was imputed by median scores.

First, the alcohol and substance use patterns and craving of abstinent versus and consuming participants were compared by either unpaired *t*-tests for continuous data or Mann–Whitney-U-tests or χ^2^ statistics for discrete data, where appropriate.

We used Kendall’s Tau b to assess correlations between negative symptoms scores and RAP and craving scores, respectively.

We compared our study sample in terms of negative symptomatology with two other subsamples consisting of patients with schizophrenia and bipolar disorder from a study by Kirschner et al. This sample is described in detail elsewhere (84). To test whether the study sample differed from patients with either schizophrenia or bipolar disorder on negative symptoms of interest, we performed oneway analysis of variance with disorder group as dependent variable and age, duration of disease, BNSS MAP, BNSS DIM and BNSS total scores as independent variables.

Finally, multiple linear regression was used to test whether depressive symptoms (CDSS total score), negative symptoms (BNSS MAP and DIM factor subscores), and alcohol drinking during the last 30 days were associated with the extent of craving (OCDS score). The conditions of linear independence, normal distribution of the dependent variable and residuals, homoscedasticity, and absence of multicollinearity (i.e., variance inflation factors all < 1.96) were met. As a goodness-of-fit measure for the model we used the adjusted R^2^ as it provides the percentage of variation explained by only the independent variables that actually affect the dependent variable.

All statistical analyses were conducted using SPSS Statistics Version 27. Given the exploratory nature of this pilot study we did not control for multiple comparisons and set the level of significance at *p* < 0.05 for all calculations.

## Results

### Demographics and sample characteristics

In total, 42 patients were included in the study and completed the clinical interviews. Detailed demographic characteristics of the sample are shown in Table 1. Over one third (*n* = 16) of the participants reached cut-off values for significant clinical depressive symptoms in the ADS-L and CDSS. Only eight participants had no psychiatric comorbidities, whereas almost half of all participants had more than one comorbid psychiatric disorder. Most patients (*n* = 35) had been formerly hospitalized more than once.

In detail, according to the MINI interview, the following comorbidities occurred within our sample: MDD: *n* = 12, dysthymia: *n* = 10, panic disorder: *n* = 10, agoraphobia: *n* = 9, social phobia = 7, generalized anxiety disorder: *n* = 10, obsessive-compulsive disorder (OCD): *n* = 1, posttraumatic stress disorder (PTSD): *n* = 9, bulimia nervosa: *n* = 3, antisocial personality disorder: *n* = 5. Consistent with the exclusion criteria, there were no patients with psychotic or bipolar disorder in our sample.

According to the MINI, more than one third (*n* = 15) of the patients fulfilled criteria for an additional substance use disorder, with cannabis use disorder being the most common (*n* = 8). sedative, hypontics and anxiolytic use disorder (*n* = 4), cocaine use disorder (*n* = 2), stimulant use disorder (*n* = 1), and opioid use disorder (*n* = 1) were also present.

Table 2 displays the current psychopharmacological medication of the study participants. There were only two participants in the sample who did not report any intake of psychopharmacological medication. Almost half of the study population (*n* = 20) had been prescribed antipsychotics; antidepressants (*n* = 30) and benzodiazepines (*n* = 22) had been prescribed to more than half of the participants. Stimulants were also prevalent in the sample (*n* = 13).

**TABLE 2:** Medication listed by substance class.

Characteristic	*N*	%
Antipsychotics	20	47.6
Opioids	4	9.5
Benzodiazepines	22	52.4
Antidepressants	30	71.4
Stimulants	13	30.9
Other	35	83.3
No medication	2	4.8

Antipsychotics: Low-potency antipsychotics predominantly have a sedative, not an antipsychotic effect. The category *Other* includes relapse prevention medication, analgesics, and medication for the treatment of somatic diseases.

### Alcohol use

The pattern of alcohol use within the sample is shown in Table 3. Of 42 participants, 38 met diagnostic criteria for AUD within the past 12 months before inclusion in the study. Two participants had shown a harmful alcohol use within the past 30 days but did not meet the diagnostic criteria of current alcohol dependence. One had been abstinent for 13 months and was thus regarded as fully remitted (9). One participant did not answer questions concerning alcohol use.

**TABLE 3:** Alcohol use and dependence pattern divided by current consumption and abstinence.

	Abstinent (*N* = 10)				Consuming (*N* = 32)				
	M	*N* (%)	SD	Range	M	*N* (%)	SD	Range	*P*
Onset harmful use	26.80		13.05	13–51	24.63		11.26	12–57	ns
Duration harmful use	15.40		11.57	2–40	19.22		12.68	1–40	ns
Severity of addiction	9.80		2.25	6–13	9.42		1.84	6–12	ns
Amount of alcohol in last 7 days in grams/in a typical week	119.93		81.26	27–301	118.80		106.81	6–392	ns
Duration abstinence in days	162.70		118.34	30–400	6.14		7.85	0–30	*
Regular consumption of other substances		4 (40)				19 (59.4)			ns
Additional substance use disorder (MINI)		4 (40)				11 (34.4)			ns
**Craving**									
VAS currently	9.50		26.40	0–84	25.16		31.50	0–100	ns
VAS 7 days	15.00		24.97	0–84	55.19		30.28	0–100	**
OCDS	14.10		9.56	4–35	20.03		8.19	4–40	ns

*p < 0.05, **p < 0.01, ns = not significant. The alcohol use and dependence pattern were collected with the substance consumption schema and craving with a Visual Analogue Scale (VAS) and the Obsessive Compulsive Drinking Scale (OCDS). Percentages are indicated in parentheses. The MINI data is missing from one participant.

Out of all participants, 10 had been abstinent from alcohol use for at least 30 days (30–400 days). Apart from the duration of abstinence, these participants did not differ significantly from the actively consuming group regarding their alcohol and substance use patterns and craving, respectively.

### Group comparison with patients with schizophrenia and bipolar disorder

Using oneway ANOVA we compared our sample with two subsamples from another study population consisting of patients either with schizophrenia or with bipolar disorder. The three groups differed significantly in regards of age and duration of disease. With respect to the extent of negative symptoms, the ANOVA revealed no significant between-group differences for BNSS total scores as well as BNSS MAP and DIM scores (BNSS total: (F(2, 91) = 1.55, *p* = 0.219, BNSS MAP: F(2, 91) = 0.26, *p* = 0.773, BNSS DIM: F(2, 91) = 2.66, *p* = 0.075).

### Negative symptoms and severity of alcohol use disorder

The BNSS total score was significantly correlated with the RAP score (τ_b_ = 0.228, *p* = 0.043, 95% CI [0.022, 0.416]). On the level of negative symptoms factors, we found a significant correlation between the BNSS MAP subscore and the RAP total score (τ_b_ = 0.223, *p* = 0.049, 95% CI [0.016, 0.411]). The DIM factor subscore of the BNSS, in contrast, did not significantly correlate with the RAP score (τ_b_ = 0.205, *p* = 0.076, 95% CI [-0.002, 0.395]). For details see Table 4.

**TABLE 4:** Correlations between BNSS MAP, BNSS DIM, BNSS Total scores, and severity of AUD (RAP), craving (OCDS), and social functioning (PSP).

			BNSS			RAP	OCDS	PSP
			MAP	DIM	Total			
BNSS	MAP	Correlation Coefficient r	–					
		p (2-tailed)	.					
		95% CI			–			
	DIM	Correlation Coefficient r			,322**	–		
		p (2-tailed)			,004	–		
		95% CI			0.123, 0.496	–		
	Total	Correlation Coefficient r	,755**	,589**	–			
		p (2-tailed)	,000	,000				
		95% CI-	0.649, 0.832	0.435, 0.710	–			
RAP		Correlation Coefficient r	,223*	,205	,228*	–		
		p (2-tailed)	,049	,076	,043	–		
		95% CI	0.016, 0.411	-0.002, 0.395	0.022, 0.416	–		
OCDS		Correlation Coefficient r	,425**	,204	,387**	,226*	–	
		p (2-tailed)	,000	,069	,000	,047	–	
		95% CI	0.239, 0.581	-0.003, 0.395	0.196, 0.550	0.020, 0.414	–	
PSP		Correlation Coefficient r	,222*	,197	,233*	,557**	,226*	–
		p (2-tailed)	,048	,086	,038	,000	,046	.
		95% CI	0.016, 0.410	–0.011, 0.388	0.027, 0.420	0.396, 0.685	0.019, 0.413	–

*p < 0.05, **p < 0.01. CI = confidence interval. BNSS MAP, Brief Negative Symptoms Scale motivation and pleasure factor; BNSS DIM, Brief Negative Symptoms Scale, diminished expression factor; BNSS Total, Brief Negative Symptoms Total Score; RAP, Rapid Addiction Profile Score; OCDS, Obstructive Compulsive Drinking Scale score; PSP, Personal and Social Performance Scale score.

The SNS total score did not show a significant correlation with the RAP score (τ_b_ = 0.201, *p* = 0.076, 95% CI [−0.007, 0.392]).

TEPS scores (total score, as well as subscores for anticipatory and consummatory anhedonia) and the CDSS total score were also not correlated with the RAP score (data not shown).

### Negative symptoms and craving

#### Non-parametric correlations

The total score of the BNSS scale was positively correlated with the OCDS total score as a measure of craving (τ_b_ = 0.387, *p* < 0.001, 95% CI [0.196, 0.550]). This was also the case for the MAP factor subscore of the BNSS (τ_b_ = 0.425, *p* < 0.001, [0.239, 0.581]). However, the DIM factor subscore did not show a significant correlation with the OCDS total score (τ_b_ = 0.204, *p* = 0.069, [−0.003, 0.395]). All data are provided in Table 4.

The SNS total score as a self-report measure for negative symptoms was significantly correlated with the OCDS total score (τ_b_ = 0.275, *p* = 0.013, 95% CI [0.072, 0.456]). TEPS scores (subscores for consummatory and anticipatory anhedonia as well as the total score), in contrast, did not show a significant correlation with the OCDS score (data not shown).

Depressive symptoms as measured with the CDSS total score were significantly correlated with the OCDS total score (τ_b_ = 0.387, *p* < 0.001, 95% CI [0.195, 0.550]).

#### Multiple regression analyses

The results obtained from the regression analysis are shown in Table 5. Multiple linear regression was used to test whether depressive symptoms. negative symptoms (MAP and DIM factor) and alcohol drinking during the last 30 days were associated with the extent of craving as measured by the OCDS. The overall regression model was significant [F(4, 37) = 9.003; < 0.001] and explained 44% of alcohol craving. with the BNSS MAP factor (β = 0.452; t = 2.78; *p* = 0.008) and number of drinking days in the last 30 days [(β = 0.233; t = 2.03; *p* = 0.049)] being significant predictors of craving. The CDSS score and the BNSS DIM factor subscore were not significantly associated with the OCDS score.

**TABLE 5:** Multiple linear regression with OCDS total score as the dependent variable and CDSS, BNSS MAP, BNSS DIM, and drinking days last 30 days as independent variables (*N* = 42).

Variable	B	SE	β	t	*p*	95% CI	
						LL	UL
Constant	6.253	2.553		2.367	0.023	0.900	11.607
CDSS (total score)	0.317	0.230	0.197	1.307	0.199	–0.175	0.810
BNSS MAP (total score)	0.388	0.133	0.452	2.783	0.008	0.106	0.671
BNSS DIM (total score)	0.166	0.164	0.128	0.983	0.332	–0.176	0.508
Drinking days last 30 days	0.802	2.325	0.239	2.033	0.049	0.003	1.601

adj. R^2^ = 0.438; F(4.37) = 9.003; *p* < 0.001. CI, confidence interval; LL, lower limit; UL, upper limit. CDSS, Calgary Depression Scale for Schizophrenia; BNSS MAP, Brief Negative Symptoms Scale. Motivation and pleasure factor; BNSS DIM, Brief Negative Symptoms Scale diminished expression factor.

### Alcohol use pattern and negative symptoms

The duration of lifetime harmful alcohol consumption as assessed *via* TLFB did not correlate with the BNSS total score (τ_b_ = 0.040, *p* = 0.712, 95% CI [−0.168, 0.245]), the BNSS MAP score (τ_b_ = 0.162, *p* = 0.139, 95% CI [−0.047, 0.357]), or the BNSS DIM score (τ_b_ = −0.084, *p* = 0.450, 95% CI [−0.286, 0.125]). The amount of alcohol consumed during the last week was also not correlated with neither the BNSS total score (τ_b_ = 0.035, *p* = 0.745, 95% CI [−0.173, 0.241]), the BNSS MAP score (τ_b_ = 0.024, *p* = 0.828, 95% CI [the BNSS MAP score 0.184, 0.230]), nor the BNSS DIM score (τ_b_ = 0.080, *p* = 0.471, 95% CI [−0.129, 0.282]).

### Social performance and negative symptoms

There was a significant correlation between the PSP total score and the BNSS total (τ_b_ = 0.233, p = 0.038, 95% CI [0.016, 0.410]), as well as the BNSS MAP score (τ_b_ = 0.226, *p* = 0.048, 95% CI [0.027, 0.420). The BNSS DIM score, in contrast, was not significantly correlated with the PSP total score (τ_b_ = 0.197, *p* = 0.086, 95% CI [−0.011, 0.388]). SNS, TEPS, and CDSS scores were not correlated with the PSP score (data not shown).

## Discussion

To our knowledge, this pilot study is the first to apply the two-factor model of negative symptoms of schizophrenia to a sample of patients with AUD.

In comparison with two samples (84) of patients with either schizophrenia or bipolar disorder, our study sample of patients with AUD showed no significant difference in the extent of negative symptoms. This finding suggests that negative symptoms that have been established as a key element of psychotic disorders are also prominent in AUD. Furthermore, there was a positive correlation between the severity of AUD as measured with the therapist-rated RAP and both the total score and the MAP factor score of the BNSS. A possible explanation is that chronic elevated alcohol use leads to changes within neural circuits that are involved in motivation and reward similar to changes that occur in schizophrenia. Diminished expression, in contrast, was not correlated to the severity of AUD.

The BNSS total score as well as the MAP factor subscore showed a significant correlation with self-reported extent of alcohol craving. While other studies have already established a correlation between anhedonia and craving (51, 67), our findings suggest that a dysfunction in a somewhat broader motivational process may be a driving factor for craving. This finding was further supported by a multiple regression analysis comparing negative and depressive symptoms in their effect on the severity of AUD, which showed that 44% of the variation of the extent of craving within our population could be explained by the BNSS MAP score and the drinking days in the past 30 days. In contrast, the DIM factor subscore was not associated with the extent of craving.

These results support the hypothesis that during the course of AUD adaptations occur withing the neural pathways involved in motivation and reward. The fact that negative symptoms within the DIM domain were not correlated with craving is in line with this interpretation.

In our sample, there was no correlation between lifetime duration of harmful alcohol use as well as the total amount of alcohol consumed, and the extent of negative symptoms. This is probably due to the small sample size. However, factors other than substance use, e.g., comorbidity or psychosocial stressors, could theoretically be responsible for the development of negative symptoms in our participants.

The findings of this pilot study are exploratory in nature and have to be replicated in other samples. If reproduced, the association of negative symptoms with severity of AUD as well as the extent of alcohol craving within these patients may have therapeutic implications. In contrast to negative symptoms in schizophrenia which are often difficult to treat (85), there is growing evidence that motivational deficits in patients with AUD can be addressed therapeutically. In a study by Kirschner et al., for example, patients with cocaine use disorder successfully activated their reward system with mental imagery and real-time fMRI neurofeedback (86). In a pilot study, Pettoruso et al. successfully treated patients with cocaine use disorder with repetitive transcranial magnetic stimulation to improve anhedonia (87). Interestingly, the NMDA receptor antagonist ketamine which has shown remarkable preliminary results in the treatment of SUDs (88, 89), also seems to have significant anti-anhedonic effects (90, 91).

The present findings should be handled cautiously. Substantial study limitations include non-random sampling, a small sample size, the absence of a comparison group, and non-adjustments for multiple testing. Notably, the majority of patients within our sample had at least one psychiatric comorbidity limiting the internal validity of our study since it cannot be ruled out that these comorbid disorders contribute to the extent of negative symptoms. However, our naturalistic sample increases the external validity since psychiatric comorbidities are the rule rather than the exception in AUD and other SUDs (92, 93). We examined the correlation of MDD and severity of AUD and craving, respectively as MDD is among the most common comorbid disorders of AUD (94) and shares anhedonia as a common feature with negative symptoms. Anxiety disorders and antisocial personality disorder were also frequent in our sample. To our knowledge, there are no studies examining the occurrence of negative symptoms in these disorders but of course our study cannot rule out a possible impact of these comorbidities on negative symptoms.

Posttraumatic stress disorder was present in nine patients. Adverse childhood events have been linked to AUD (95, 96) as well as positive and negative symptoms in schizophrenia (97). Future research should investigate the nature of a possible interrelation between PTSD and negative symptoms in AUD. We further used a cross-sectional approach and did not assess important parameters, such as an objective measure of alcohol intake, a valid measure for the severity of AUD. Furthermore, the RAP that we used to assess overall severity of AUD, which is a reliable clinical tool, has not yet been validated in other studies.

Taken together, however, our findings provide first evidence that negative symptoms are prevalent in AUD to an extent that does not differ significantly from other major psychiatric disorders, are correlated with disease severity and craving, and therefore might constitute a novel and promising therapeutic target that should be addressed in future clinical trials to improvement treatment outcomes for patients with AUD.

## Data availability statement

The raw data supporting the conclusions of this article will be made available by the authors, without undue reservation.

## Ethics statement

The studies involving human participants were reviewed and approved by Cantonal Ethics Committee, Zurich, Switzerland. The patients/participants provided their written informed consent to participate in this study.

## Author contributions

MB, KD, and MH designed the study with assistance from MK and SK. GF and JH performed all the clinical interviews and collected all therapist-rated measurements. MK instructed GF and JH in the assessment of all negative symptom scales. GF, JH, KD, and MB analyzed the data. MB wrote the manuscript with input from all authors. All authors contributed to the article and approved the submitted version.
